# Sleep Quality and Sleep Disturbance Perception in Dual Disorder Patients

**DOI:** 10.3390/jcm9062015

**Published:** 2020-06-26

**Authors:** Gianina Luca, Lola Peris

**Affiliations:** CNP-Centre Neuchâtelois de Psychiatrie, 2074 Marin, Switzerland; gianina.luca@cnp.ch

**Keywords:** dual disorders, addiction, sleep, risk factors

## Abstract

Background: Sleep problems are particularly frequent in psychiatric disorders, but their bidirectional intersection is poorly clarified. An especial link between substance use and sleep seems to exist. While dual disorder patients are certainly at higher risk of experiencing sleep problems, very limited research is available today. Methods: Forty-seven dual disorder hospitalized patients were included in this first study. A complete psychiatric evaluation was performed, and sleep habits, patterns and potential disorders were evaluated with specific sleep scales, as well as anxiety. Results: The global prevalence of insomnia symptoms was considerably higher compared with the general population. Different abuse patterns as a function of concurrent psychiatric diagnosis were found, with no significant gender differences. The association between the investigated sleep parameters and any specific substance of abuse was minor. The addict behavior started in more than half of the patients prior to the main psychiatric diagnosis and close to the beginning of sleep problems. Men had a higher prevalence of insomnia symptoms, together with a higher incidence of anxiety. Overall, subjective daytime functioning was not altered as a consequence of poor sleep. Conclusion: Dual disorder patients face significant sleep disturbances, with low sleep quality. The role of sleep in addiction and dual disorders deserves greater research.

## 1. Introduction

Sleep problems are especially frequent in psychiatric disorders, but their complex bidirectional interactions are still poorly understood and, although several common neurobiological abnormalities may explain why sleep disorders are related to the risk of developing different psychiatric disorders, the causal relationships have not been clearly identified [1,2]. Xue Gao et al. noted the work of Charrier et al., stressing that the single-nucleotide polymorphisms in the core circadian genes are associated with psychiatric disorders [3], and of Akers et al., proposing methods for using sleep as a therapy for psychiatric disorders after evaluating the regulation of sleep and epigenetic modifications of adult neurogenesis and memory consolidation [2,4].

In a large community study with a one-year follow-up interview evaluating sleep disturbances and psychiatric disorders, Ford and Kamerow noted a much higher prevalence of psychiatric disorders in those with sleep complaints, as well as a greater rate of new psychiatric disorders one year later, also among those with sleep complaints [5]. A cross-sectional (six-month) association between sleep disturbances and major depression, anxiety disorders and substance use disorders is reported in this study. Similar findings were described by Breslau et al. in a longitudinal epidemiological study of young adults [6], while Xue Gao et al. suggested the causal role of insomnia in autism spectrum disorder and bipolar disorder [2] and Acker et al. stated the relevance of psychiatric comorbidities in the treatment of patients with obstructive sleep apnea syndrome, focusing their research on depression [7].

The relationship with addiction and dual disorders is probably the strongest example of this interaction. It has long been known that sleep problems are more prevalent among persons with use of or dependence on substances and that those with sleep problems have a higher risk of developing substance use problems than the general population [5,8]. Nevertheless, although evidence links sleep and substance use, little research exists on this topic.

Overall, it is estimated that between 50% and 80% of the treatment-seeking psychiatric population is affected by insomnia. People with substance use disorders (SUD) are at an especially high risk of suffering sleep problems. For example, between 36% and 72% of alcohol-dependent patients treated in addiction facilities report significant sleep problems [9,10].

Sleep problems in SUD may appear as direct effects of the substance or as a result of withdrawal, but pre-existing sleep problems may also provoke the development of addiction [10,11].

In a cross-sectional study utilizing retrospective self-reported sleep disturbance and substance use, Dolsen and Harvey found that a lifetime history of insomnia and hypersomnia was associated with a higher frequency of all substance use at treatment entry and higher rates of cocaine use at 12-month post-treatment assessment, although the characteristics of the study did not allow it to define if sleep disturbances were caused by substance use, if substance use caused sleep disturbances or if other variables could influence these relationships [12].

Most of the drugs of abuse are disruptive to sleep and/or daytime alertness, but such disturbances are not major criteria for SUD in current psychiatric classifications [13]. Cannabis, sedative hypnotics and alcohol may become reinforcers and lead to substance use given their capacity to induce sleep in persons with insomnia. Further, risky patterns of substance use are associated with poor sleep, potentially inducing a risk of future mood or other psychiatric disorders and/or poor levels of functioning [14]. The alerting effects of stimulants are reinforcing for individuals who experience sleepiness, fatigue or have difficulty functioning at a “normal” level. Rotating shift workers and night workers report a disproportionate use of sedative drugs, especially alcohol to improve their sleep and stimulants, and especially caffeine to improve their alertness: this kind of substance use may increase risks of misuse. Similarly, acute rapid eye movement (REM) sleep deprivation by awakening enhances pain sensitivity, according to studies. As opiates suppress REM, their analgesic effect may be reduced. Whether this hypothesized reduction leads to a need for higher doses or to the development of physical dependence is also a critical issue [13].

Abnormal sleep patterns can persist for up to three years in alcohol dependence. However, while it is tempting to attribute these sleep abnormalities to excessive alcohol drinking, sleep problems could precede the development of dependence, or they could be secondary to the development of other medical or psychiatric disorders related to excessive alcohol drinking. Some studies suggest that a slow wave sleep deficiency may be associated with the development of alcohol dependence (it is known that alcohol enhances slow wave sleep with acute use) [13].

It has been hypothesized that continued substance use, difficulty reducing use and relapse may reflect “self-medication” to reverse the excessive sleepiness when abstaining from some substances. Both the objective and subjective measures of sleep after acute abstinence predict the likelihood of relapse during long-term abstinence better than variables such as age, employment, marital status, severity of alcoholism, hepatic enzymes or depression ratings. In cocaine-dependent individuals, total sleep time was positively related to days of abstinence over a six-week study. These data raise the question whether insomnia-focused treatment would have a beneficial effect on substance use treatment. Although the role of sleep/alertness disturbances in SUD is not fully understood, the need for clinical trials that focus on the treatment of sleep complaints in substance use is clearly evident. Sleep disturbance seems to be causally related to alcohol and drug use, either as the precipitant or the consequence. Sleep disturbance may be comorbid (as suggested in the literature), and treatment must be directed at both disorders, independent, or related to a third common factor [13,15].

Insomnia and SUD may share common neurobiological disturbances. Data suggest that insomnia is a disorder of “hyperarousal”, which constitutes, in part, HPA-axis dysfunction involving corticotropin releasing hormone (CRF) and norepinephrine (NE). Many theories of addiction hypothesize that stress increases one’s vulnerability to drug use. The activation of the brain circuits involved in stress lead to CRF/NE activation, which also activates the dopaminergic brain motivational pathways known to be engaged by drugs of abuse [13]. Alterations in the dopaminergic neurons have long been associated with alcohol and drug use but also with other disorders like post-traumatic sleep disorder and autism. The causal role of mesocorticolimbic dopaminergic activity in sleep and wakefulness has been described by authors like Eban-Rotschild et al. [16].

The orexin system is thought to have a role in the rewarding effects of drugs, in addition to its role in arousal. It has been suggested that orexin is specifically engaged in substance use during elevated motivational states, such as when the effort to obtain the drug is high or when stress exists. Orexin antagonists like suvorexant, which is currently approved for the treatment of insomnia, are being evaluated for the treatment of SUD, as the effect of orexin could be of help in attenuating drug rewards and improving sleep disturbances by preventing the potentiation of reward and arousal [17,18].

To fully identify how the circuits and substrates that regulate sleep and arousal intersect with those that mediate rewards and how they are targeted by drugs should be the “first step” to advance in this field. However, although the neurobiological links between sleep dysfunction and substance use behavior are well accepted, the research is still in its nascent stages. Moreover, how the interaction between sleep and substance use is modulated by genetics, life events, sex and circadian rhythms remains largely unknown [16]. Nevertheless, research is increasingly showing that sleep disturbances experienced early in life may precede and/or predispose an individual to develop SUD, and a growing number of studies support the bidirectional component of the relationship between sleep patterns and substance use [19].

Roncero et al. [20] found that almost 70% of drug-dependent in-patients reported sleep problems prior to admission and that 80% of those patients related such problems to their substance use. Nevertheless, according to Arnedt et al. [8], in clinical settings, there may be a failure to appreciate the importance of sleep, especially in the early stages of recovery from addiction. Untreated sleep problems substantially increase the likelihood of relapse for those addicted to drugs.

Gender and age are the most important demographic variables related to the prevalence of insomnia, with women experiencing a higher rate of insomnia than men and complaints of insomnia increasing with age (although this association seems not to be robust when more severe criteria of insomnia are applied) [21,22]. Medical and psychiatric status influence the frequency of insomnia, which is more frequent among patients with psychiatric disorders. Moreover, very few studies address the question of the interaction between addiction, sleep and sex. One of them, evaluating problematic internet use and risk of sleep disturbances in adolescents, found a greater association in girls [23]. In addition, according to the study of Ogeil et al. [24], women reporting risky alcohol and cannabis use had the highest global Pittsburgh Sleep Quality Index scores, reflecting the poorest sleep quality.

Gender differences in mental disorders are well documented. However, although some studies have detected differences for dual disorders (DD) [25,26], little published research is available to establish clear conclusions. However, prevention, treatment, and prognosis would undoubtedly benefit the individualization of each gender characteristic for DD.

Published studies in this field have mainly referred to psychiatric patients or substance use disorder patients, indicating only marginally the possibility of co-occurring disorders among some of those patients. Evidence suggests that DD are the rule rather than the exception. The interaction between a sleep problem, a substance use disorder and another psychiatric disorder certainly adds more complexity.

Bearing this problem in mind, we decided to develop the first observational study to characterize potential sleep problems in a sample of hospitalized dual disorder patients. Although several studies have focused globally on sleep and SUD, this preliminary cross-sectional study was directly focused on sleep and DD patients, aiming to investigate the frequency and severity of sleep disturbances one month prior to admission for on-demand controlled withdrawal.

## 2. Materials and Methods

### 2.1. Sample

Forty-seven patients with dual diagnosis hospitalized in our dual disorders unit (Préfargier Hospital, Marin-Epagnier, Switzerland) were randomly invited to take part in the study after obtaining their informed consent. Four of them (2 men, 2 women) refused to participate. The project was approved by the hospital and was carried out according to the directives of the Swiss Federal Office of Public Health and following the rules of the Declaration of Helsinki.

Patients were admitted electively, at their request, for controlled withdrawal. A tailored treatment plan was discussed with the participants prior to hospitalization. This project included treatment settings, discussion of psychopharmacologic approach, participation in different groups and assessment of personal needs (social services, family or couple therapy, occupational therapy, long term rehabilitation institution projects, etc.).

A complete psychiatric evaluation was performed at admission by the medical team of the unit. Psychiatric diagnoses were established according to the International Classification of Diseases, ICD-10 criteria [27]. None of the patients presented an acute exacerbation of their main psychiatric disorder.

Beside the specific complete psychiatric evaluation, after the resolution of withdrawal symptoms, patients were invited to complete a comprehensive set of self-administered questionnaires (Supplementary Figure S1) evaluating their sleep habits and sleep patterns, as well as specific sleep disorder questionnaires.

The Pittsburgh Sleep Quality Index (PSQI) is a validated, effective, self-administered questionnaire used to evaluate sleep quality over the last 30 days. It has seven domains (regrouped from 19 questions), each rated from 0 to 3 (sleep duration, sleep efficiency, sleep latency, sleep disturbance, daytime dysfunction, frequency of sleep medications and subjective sleep quality), where 3 is the negative extreme. The cut-off score of 5 allows the distinction between healthy controls and persons with poor sleep quality. [28]

The Insomnia Severity Index (ISI) is a 7-item self-report questionnaire assessing perceived insomnia severity over the last month. This questionnaire evaluates the severity of sleep onset, sleep maintenance and early morning awakening problems, as well as the impact of these disturbances on daytime functioning. The total score ranging from 0 to 28 includes four categories: absence of insomnia (0–7), sub-threshold insomnia (8–14), moderate insomnia (15–21) and severe insomnia (22–28) [29].

Regarding PSQI and ISI, patients were asked to consider the month before admission, in order to avoid the bias of sleep disturbances during the withdrawal process.

The Epworth sleepiness scale (ESS) is widely used to assess sleepiness (in the general population and in subjects with sleep disorders). This scale rates the chance of falling asleep in eight different situations. A score higher than 10 is associated with excessive daytime sleepiness [30].

In order to evaluate the presence of an intrinsic sleep disorder as central hypersomnia, obstructive sleep apnea syndrome or restless legs syndrome, the clinical interview focused on related symptoms. The set of questions was derived from the International Classification of Sleep Disorders, third edition [31]. The presence of insomnia was based on actual diagnostic criteria: difficulties initiating or maintaining sleep impacting daytime functioning (fatigue, impaired social, family, occupational or academic performance, daytime sleepiness, etc.) [32] which cannot be explained purely by inadequate opportunity; the sleep disturbance and associated daytime symptoms occur at least three times per week and have been present for at least 3 months.

Furthermore, the potential existence of anxiety was evaluated with the GAD-7 [33], a valid and efficient tool for screening generalized anxiety disorder and assessing its severity in clinical practice and research. Mild, moderate and severe anxiety are established based on the three cut-off scores of 5, 10 or 15 points out of a maximum of 21.

### 2.2. Sleep Disturbances

The presence of sleep disturbances was considered based on the results obtained in the above-described questionnaires: PSQI > 5 or ISI > 14 or ESS > 10. One patient was previously diagnosed and successfully treated for respiratory disturbances during sleep.

### 2.3. Other Variables

Data on demographic and functional variables were obtained: ethnicity (Caucasian/other, with Caucasian group as reference) marital status (married, divorced, never married, with “married” group as reference), employment status (employed/unemployed, with “employed” group as reference) and academic achievement.

Less than 9 years of school was considered low academic achievement, normal academic achievement was considered completing high school and high academic achievement meant a bachelor’s degree or higher. For the analysis, we used “normal academic achievement” as the reference group.

These data were obtained from admission records and confirmed through the addressed questionnaires.

Patients gave detailed information regarding the primary drug of concern. We also collected data about the patients’ comorbid psychiatric and medical conditions, actual medications and duration of the admission.

### 2.4. Statistical Analysis

Data collected from each patient were completely anonymized. All statistical tests were performed using SPSS 22.0, (IBM SPSS Statistics, Armonk, NY, USA). Gender differences in the distribution of sleep disturbances and sleep quality were tested for significance by using a χ²-test. Given the small sample size, we split the patients into three groups based on the most commonly reported drugs of concern: cannabis only, alcohol only and multiple psychoactive substances. In our sample, none of the patients declared using stimulants. On the same basis, we created three diagnostic groups to classify co-occurring psychiatric conditions according to the main psychiatric diagnosis identified in our sample: affective disorders, psychotic disorders and personality disorders.

Differences in the demographic and clinical variables between genders were examined by using a Fisher’s exact test and a *t*-test for independent samples. The corresponding χ² values, odds ratios and 95% confidence intervals (CI₉₅) are reported. Furthermore, logistic regression was also employed to test binary variables. Initiation insomnia (binary variable, presence of insomnia as reference) was defined as the presence of difficulties falling asleep occurring more than 3 nights/week, when normal sleep conditions are present. Superficial (or fragmented) sleep was defined as the feeling of frequent awakenings or shallow sleep (presence of fragmented sleep as reference). Maintaining insomnia was defined as difficulties in maintaining sleep or early morning awakenings (presence of these symptoms as reference). Beginning of sleep disturbances was classified as before or after the initiation of substance abuse (before as reference).

The categorical variables were analyzed using categorical factor analysis. Principal component analysis for categorical data allowed us to analyze the probable association between categorical data. In the second step, to assess the main factor associated with sleep perception, we separately analyzed the data for men and women through a multivariate regression analysis.

## 3. Results

### 3.1. Demographics

Out of the 43 patients (mean age 42.6 ± 10.2 years old), 68% were male and all but one (African, cannabis user) were Caucasians. The demographic data are presented in Table 1.

Most cannabis and alcohol users had a normal education level, while multiple substances users had a lower education degree. We found no association between alcohol use and the demographic data (see Table 1).

### 3.2. The Substance of Use and Co-Occurrent Psychiatric Disorders

All included patients used at least one substance of abuse or dependence according to the International Classification of Diseases, ICD-10 criteria. We found different use patterns as a function of co-occurrent psychiatric diagnoses (χ² 24.495, *p* < 0.001). The preferred substance of use was cannabis for 15.9% of patients, 66.6% with a diagnostic of a psychotic disorder. Alcohol was the substance of use for 43.2% of patients, 63.2% with a co-occurrent affective disorder. Polysubstance users mainly had a personality disorder diagnosis (58.8%). Table 2 presents the distribution of substance of use as a function of co-occurrent psychiatric disorder.

No gender differences were found for the association between the primary substance of concern and psychiatric diagnosis (multivariate regression analysis, Supplementary Table S1)

### 3.3. Psychiatric Diagnosis and Medication

The mean reported duration since the first main psychiatric diagnosis was 12.4 +/− 3.2 years, with no significant differences between men and women. The mean reported duration of psychoactive substance use was 14 +/− 2.4 years (data from 23 patients only).

In our sample, more men than women had a personality disorder (*p* = 0.10). The anxiety symptoms evaluated by GAD-7 were more intense and more frequent among the men (χ²-test, *p* = 0.04).

In total, 62.8% of patients had experienced psychotropic treatment before admission, and 37.2% took sleep-inducing medication, which continued during the withdrawal period. The mean duration of the actual admission was 20.4 days.

Half of the patients had had at least one relapse to their substance of use, and 25.6% were hospitalized for decompensation of their main psychiatric disorder in the past two years.

### 3.4. Sleep Characteristics

Half of the patients declared having sleep difficulties for more than 10 years, with problems implementing coping strategies. The main reported sleep problem was difficulty falling asleep, with cannabis and alcohol users declaring higher sleep onset latency compared with polysubstance users (53.4 ± 5.3 and 51.2 ± 3.2, respectively, versus 40.2 ± 5.3 minutes).

In total, 71.5% of cannabis users and 68.4% of alcohol users declared starting their substance use after the development of insomnia, while for most polysubstance users (64.7%), drug use was not related to the development of sleep disturbances (χ²-test 5.46, *p* = 0.046).

Most of the patients (69%) declared fragmented or superficial sleep. Persistent insomnia symptoms were present in 63.3% of patients, with a trend toward a higher frequency among patients with depression as their main psychiatric diagnosis (*p* = 0.1).

Men had higher risk for developing initiation insomnia symptoms (Table 3) and this risk increased even more after adjusting for psychiatric disorder and substance of abuse, while women were more exposed to maintaining insomnia symptoms. After adjusting for demographic covariates (education, marital status or employment), these results are no longer significant.

Overall, the mean subjective sleep duration was diminished (6.05 ± 0.22 hours) and reported sleep latency increased (48.3 ± 3.2 minutes). Consequently, sleep efficiency was slightly reduced (84.1 ± 9.3%.). The crude unadjusted analysis showed that women declared better sleep quality and a better daytime functioning than men did, with the latter association close to statistical significance (see Table 4 for details), although these differences disappeared after controlling for confounders (substance of use, main psychiatric diagnosis and age) (Supplementary Table S2).

In our sample, 71.5% of cannabis users and 68.4% of alcohol users declared starting the substance use after the development of insomnia, equally associating the use of these substances as to correct their initiation insomnia. After controlling for multiple confounding factors (age, gender and psychotropic medication), this association was no longer statistically significant.

For all patients but two, the Epworth sleepiness scale score was inferior to 10 points (8.4 ± 2.4).

### 3.5. PSQI Domains

In our sample, the total PSQI score was altered in both genders (mean 9.8 +/− 4.21) with no differences between men and women (*t*-test, independent samples, *p* = non significant (ns), with 74% of the patients having a PSQI score higher than 5.

The domain analysis showed no gender differences in any of the measures except for sleep quality and the presence of anxiety (Table 4). Although not statistically significant, we noted a trend for lower subjective sleep duration and more altered daytime functioning in men (Table 4).

Patients reporting insomnia symptoms had a higher PSQI score, had lower sleep duration (Table 5) and more frequently used sleep medication (OR 3.2, CI₉₅ 0.7 to 5.6).

Cannabis users reported more frequently impaired sleep onset latency compared with multiple substance users (OR 1.58, CI₉₅ 0.14 to 2.80), while alcohol use was associated more frequently with initiation and maintaining sleep difficulties compared with the same group (OR 2.90, CI₉₅ 0.25 to 5.70), even if statistical significance disappeared after correcting for confounding factors.

Interestingly, subjective daytime functioning was, overall, not altered as a consequence of poor sleep (χ² 4.30, *p* = 0.23).

The principal component analysis for categorical data run on sleep and main psychiatric diagnosis variables retained six dimensions explaining 79.16% of the variability (Supplementary Table S3). The first dimension loaded sleep medication use, presence of sleep disturbances, difficulties falling asleep and low sleep quality. Cannabis use associated with altered sleep quality and increased sleep onset latency. Alcohol and cannabis use loaded on the fourth dimension, together with superficial sleep, and diminished sleep duration and increased sleep latency. Intriguingly, multiple substance use was associated with better sleep efficiency. Initiation and maintaining insomnia loaded with alcohol use.

In a second step, we looked at demographic variables which may impact the results and this analysis retained two major dimensions with a significant eigenvalue (>1) suggesting that men had more severe insomnia symptoms, were more anxious and had a higher total PSQI score (Supplementary Table S3).

## 4. Discussion

This preliminary study subjectively assessed patient sleep patterns one month prior to on-demand admission for controlled withdrawal in the absence of decompensation of the main psychiatric diagnosis.

The distribution of the primary substance of use related to the psychiatric diagnoses in our sample was consistent with the data previously published in the literature.

The global prevalence of insomnia symptoms was significantly higher compared with the general population and also congruent with the data described previously [31,34]. While insomnia prevalence in the general population is higher among women, the higher prevalence of insomnia symptoms among the men in our sample could be associated with their higher incidence of anxiety. As expected, anxiety is highly associated with sleep initiation difficulties.

As already suggested previously [10], the association between the investigated sleep parameters and the specific substance of use was minor, indicating that disturbed sleep is highly prevalent among patients using a substance, regardless of the type of substance, although those consuming alcohol or cannabis declared increased sleep onset latency.

Interestingly, patients using multiple substances (almost 60% of them with a personality disorder diagnosis) did not associate the use of substances or relapse with sleep difficulties.

It is important to note that none of our patients used stimulants, which may impact sleep in a different manner. While before testing the effect of potential confounding variables, cannabis and alcohol users declared altered sleep, the polysubstance users did not. The lack of the association between sleep disturbances and multiple substance use may be due to the effect of the different substances used masking the presence of sleep initiation or maintaining difficulties.

Even though it is generally assumed that chronic altered sleep quality could potentiate the impact of substance use on cognitive function [35], the absence of daytime functioning alteration by poor sleep in our sample is a puzzling issue worth exploring further. Equally, although the data relating substances of use to an underlying psychiatric diagnosis are globally in line with those already published (likewise for the higher prevalence of sleep problems compared with the general population), the predominance of insomnia symptoms in men together with the higher presence of anxiety in this group warrant further study.

As addictive behavior was engaged in more than half of the patients prior to the onset of their sleep problems, we maintain the bidirectional link between sleep disturbances and the use of psychoactive substances. Due to the cross-sectional nature of this study, it is impossible to elaborate more on this relationship here. The complex factors moderating/mediating this bidirectional relationship deserve further investigation.

Several limiting factors affect the results of this study:

First, the sample size is small compared with the high variability of main psychiatric diagnosis and substance of use. Fourteen patients had a personality disorder diagnosis (four borderline, four antisocial, three avoidant and three dependent), and a small number of patients in each category did not permit a detailed analysis.

Moreover, patients did not fill in questionnaires about the severity of their addiction one month prior to admission, and this factor could have added significant information.

Finally, the retrospective design of the study, based mainly on patient reports, does not allow a more objective evaluation of certain data, especially the chronologic appearance of the disorders (as the diagnostic delay for some psychiatric troubles is well known). A recall bias in collecting subjective data due to the nature of the study and of the administered questionnaires could have impacted the results.

Due to the high variability of the clinical status of patients for a better analysis, future inclusion criteria should be more restrictive, and a bigger sample should be included.

## 5. Conclusions

Independently of the main psychiatric diagnosis and other parameters, dual disorder patients face significant sleep disturbances with low sleep quality. Implementing detailed sleep evaluations and proposing different strategies to diminish such sleep problems seem to be highly important in the evolution of these patients. Further studies and follow-up studies could offer an answer to the role of sleep in addiction and dual disorders and support the importance of a good quality of sleep, together with stabilizing the main psychiatric disorder, in diminishing the frequency or the severity of the use of psychoactive substances among these patients.

While still in its beginnings, research advances on sleep disturbances in the context of substance use and dual disorders may greatly improve the knowledge of substance use disorders etiology and, therefore, find new methods of prevention and treatment.

## Figures and Tables

**Table 1:** Demographic characteristics of the sample by gender and primary substance of concern. Results are presented as number and percentage (%). Significant χ²-test results are marked in bold. OR- Odds ratio

	Cannabis Users N (%)			Alcohol Users N (%)			Multiple Substances Users N (%)		
	All	Men	Women	All	Men	Women	All	Men	Women
Marital status - married	3 (7.5%)	1 (3.4%)	2 (15.4%)	9 (22.5%)	7 (25.9%)	2 (15.4%)	6 (15.0%)	5 (18.5%)	1 (7.7%)
Marital status - divorced	2 (5.0%)	2 (6.9%)	0	7 (17.5%)	4 (11.1%)	3 (22.6%)	7 (17.5%)	5 (18.5%)	2 (15.4%)
Marital status - never married	2 (5.0%)	1 (3.4%)	1 (7.7%)	3 (7.5%)	2 (7.4%)	1 (7.7%)	4 (10.0%)	2 (7.4%)	2 (15.4%)
Employment status - employed	3 (6.9%)	2 (6.9%)	1 (7.1%)	11 (25.5%)	10 (34.4%)	1 (7.1%)	7 (16.2%)	6 (20.7%)	1 (7.1%)
Employment status -unemployed	4 (9.3%)	2 (6.9%)	2 (14.3%)	8 (18.6%)	3 (10.3%)	5 (35.7%)	10 (23.2%)	6 (20.6%)	4 (28.5%)
Academic achievement - low	0	0	0	1 (2.3%)	0	1 (7.1%)	6 (13.9%) *	5 (17.2%)	1 (7.1%)
Academic achievement - normal	6 (13.9%)	4 (13.7%)	2 (14.2%)	15 (34.8%)	11 (37.9%)	4 (28.5%)	11 (25.5%)	7 (24.1%)	4 (28.5%)
Academic achievement - high	1 (2.3%)	0	1 (7.1%)	3 (6.9%)	2 (6.9%)	1 (7.1%)	0	0	0

*chi squared 7.45; OR 13.64, *p* = 0.006

**Table 2:** Substance abuse by co-occurrent psychiatric disorder. Unadjusted odds ratios and 95% confidence intervals are presented. Significant results are marked in bold.

Primary Drug of Concern	Diagnosis	Substance Use (%)	No Substance Use (%)	Odds Ratio	95% Confidence Interval
Cannabis	Psychotic disorder	57.1	5.5	**22.67**	2.86–179.18
	Affective disorder	28.5	58.3	0.29	0.04–1.67
	Personality disorder	14.3	36.1	0.29	0.03–2.72
Alcohol	Psychotic disorder	0	25	**0.07**	0.01–1.38
	Affective disorder	84.2	29.1	**12.95**	2.84–58.92
	Personality disorder	15.8	45.8	**0.22**	0.05–0.96
Multiple substances	Psychotic disorder	11.8	15	0.73	0.11–4.52
	Affective disorder	29.4	69.2	**0.19**	0.04–0.70
	Personality disorder	58.8	15.4	**7.86**	1.86–33.09

**Table 3:** Gender effect on insomnia and the onset of sleep complaints.

	OR	95% Confidence Interval	
		Lower	Upper
Initiation insomnia			
Unadjusted model	5.16	1.134	23.494
Model 1	9.71	1.47	64.08
Model 2	0.00	0	.
Maintaining insomnia	0.23	0.003	0.988
Unadjusted model	0.04	0.001	1.005
Model 1	0.16	0.022	1.201
Model 2	0.00	0	
Superficial sleep or frequent awakenings			
Unadjusted model	0.29	0.059	1.454
Model 1	0.16	0.022	1.201
Model 2	0.00	0	
Onset of sleep disturbance after onset of SUD			
Unadjusted model	2.28	0.548	9.517
Model 1	2.57	0.464	14.268
Model 2	0.00	0	

Logistic regression analysis (female gender as reference). Model 1 is adjusted for substance of use, main psychiatric diagnostic and the presence of anxiety. Model 2 is adjusted for substance of abuse, main psychiatric diagnostic, the presence of anxiety and socio-demographic variables (marital status, employment status, education). OR—odds ratio; SUD—substance use disorder.

**Table 4:** Gender impact on analyzed variables ¹.

	All	Men (*n* = 29)	Women (*n* = 14)	*p*-Value
Age ¹		40.65 (9.78)	45.64 (10.8)	0.158
Epworth ¹		9.10 (4.1)	6.73 (3.2)	0.526
Sleep onset latency (min) ¹	48.3 (3.2)	43.77 (21.7)	50.46 (28.37)	0.158
Sleep duration (hours) ¹	6.06 (0.22)	6.01 (1.54)	6.26 (1.75)	0.663
Sleep efficiency (%) ¹	84.1 (9.30)	82.36 (14.01)	86.83 (12.95)	0.702
PSQI-total score ¹	9.84 (4.2)	10.54 (3.85)	8.3 (4.73)	0.209
PSQI domains ²				
Sleep disturbances - 0	9.30%	2.33%	6.98%	
Sleep disturbances - 1	34.88%	23.26%	11.63%	
Sleep disturbances - 2	41.86%	30.23%	11.63%	
Sleep disturbances - 3	13.95%	11.63%	2.33%	0.244
Sleep onset latency - 0	19.51%	9.76%	9.76%	
Sleep onset latency - 1	31.71%	21.95%	9.76%	
Sleep onset latency - 2	41.46%	31.71%	9.76%	
Sleep onset latency - 3	7.32%	4.88%	2.44%	0.621
Daytime dysfunction - 0	20.93%	11.63%	9.30%	
Daytime dysfunction - 1	32.56%	20.93%	11.63%	
Daytime dysfunction - 2	34.88%	25.58%	9.30%	
Daytime dysfunction - 3	11.63%	9.30%	2.33%	0.067
Sleep efficiency - 0	47.22%	30.56%	16.67%	
Sleep efficiency - 1	16.67%	8.33%	8.33%	
Sleep efficiency - 2	22.22%	22.22%	0	
Sleep efficiency - 3	13.89%	11.11%	2.78%	0.159
Sleep medication - 0	20.93%	13.95%	6.98%	
Sleep medication - 1	18.61%	6.98%	11.63%	
Sleep medication - 2	30.23%	20.93%	9.30%	
Sleep medication - 3	30.23%	25.58%	4.65%	0.169
Sleep quality - 0	25.58%	6.98%	18.61%	
Sleep quality - 1	23.26%	9.30%	13.95%	
Sleep quality - 2	30.23%	18.61%	11.63%	
Sleep quality - 3	20.93%	16.28%	4.65%	0.04
Sleep duration - 0	35.90%	20.51%	15.39%	
Sleep duration - 1	10.26%	5.13%	5.13%	
Sleep duration - 2	35.90%	33.33%	2.56%	
Sleep duration - 3	17.95%	7.69%	10.26%	0.06
Insomnia – present ²	63.46%	44.19%	18.28%	0.332
Anxiety symptoms – present ²	88.23%	70.58%	17.64%	**0.019**

Continuous variables were analyzed using t-test for independent samples; ² categorical variables were tested using chi-squared test. Significant results are marked in bold.

**Table 5:** Impact of insomnia symptoms on subjective continuous sleep parameters.

Variable	Insomnia Symptoms	Mean Difference	95.00% Confidence Interval		*t*	df	*p*-Value
			Lower Limit	Upper Limit			
PSQI score	absent	−4.4	−7.08	−1.72	−3.354	30	**0.002**
	present						
PSQI sleep onset latency (min)	absent	−23.4	−37.5	−9.29	−3.359	38	**0.002**
	present						
PSQI sleep efficiency	absent	2.19	−7.02	11.42	0.485	32	0.631
	present						
PSQI sleep duration (h)	absent	0.91	−0.12	1.95	1.787	37	0.082

Results from a *t*-test for independent samples. Significant *p* values are marked in bold. PSQI: Pittsburgh Sleep Quality Index.

## Supplementary Materials

The following are available online at https://www.mdpi.com/2077-0383/9/6/2015/s1, Table S1: Multivariate regression analysis, model adjusted for gender and gender interaction; Table S2: Multivariate regression: multivariate analysis estimating diagnostic impact on analyzed parameters—fully adjusted model; Table S3—CATPCA—principal components analysis for categorical data; Figure S1. Time line of data collection.
