# Supplementary material for: Sleep Quality and Sleep Disturbance Perception in Dual Disorder Patients
Source: J Clin Med. 2020 Jun 26;9(6):2015. doi: 10.3390/jcm9062015 (PMC7355436; doi:10.3390/jcm9062015)
Supplement: Supplementary file 1 [file jcm-09-02015-s001.pdf]

Supplementary table 1 - Multivariate regression analysis, model adjusted for gender and gender interaction.

Parameter	Odds Ratio	95% Confidence Interval	
		Lower	Upper
**Choice Group: Cannabis vs Multiple substances use**			
Gender by psychotic disorders diagnosis	0	0	0
Gender by affective disorders diagnosis	1.5	0.024	92.25
Gender	0.167	0.02	1.384
Psychotic disorder diagnosis	45740	45740	45740
Affective disorder diagnosis	1	0.063	15.988
Choice Group: Alcohol vs Multiple substances use			
Gender by psychotic disorders diagnosis	0	0	0
Gender by affective disorders diagnosis	4.5	0.174	116.084
Gender	0.167	0.02	1.384
Psychotic disorder diagnosis	0	0	0
Affective disorder diagnosis	4	0.447	35.788

Supplementary table 2. Multivariate regression: multivariate analysis estimating diagnostic impact on analyzed parameters – fully adjusted model.

		95% Confidence Interval	
	OR	lower	upper
Threshold [psychotic disorder]	59.06	-2779.73	2661.61
Location gender	13.34	-4608.20	4634.89
[anxiety=0]	53.01	-10425.85	10319.82
[anxiety=1]	0a		
[sleep duration=0]	53.66	-8501.34	8608.67
[sleep duration=1]	80.78	-12058.26	12219.83
[sleep duration=2]	77.74	-10965.18	11120.67
[sleep duration=3]	0a		
[sleep disturbances=0]	58.41	-6655.83	6539.00
[sleep disturbances=1]	113.76	-11406.71	11179.18
[sleep disturbances=2]	43.73	-3643.01	3555.54
[sleep disturbances=3]	0a		
[sleep latency=0]	6.10	-2367.67	2379.88
[sleep latency =1]	45.44	-2745.88	2654.99
[sleep latency =2]	24.35	-2447.06	2398.36
[sleep latency =3]	0a		
[daytime dysfunction=0]	36.14	-5340.61	5268.32
[daytime dysfunction=1]	87.64	-9642.56	9467.27
[daytime dysfunction=2]	111.39	-14175.64	13952.85
[daytime dysfunction=3]	0a		
[sleep efficiency=0]	78.10	-5227.31	5383.52
[sleep efficiency=1]	110.86	-10213.86	10435.59
[sleep efficiency=2]	86.41	-7744.54	7917.37

[sleep efficiency=3]	0a		
[sleep quality=0]	40.96	-4873.31	4791.37
[sleep quality=1]	29.06	-2377.11	2435.24
[sleep quality=2]	39.76	-7312.93	7392.46
[sleep quality=3]	0a		
[sleep medication=0]	85.81	-13079.01	13250.65
[sleep medication=1]	0.91	-3849.09	3850.92
[sleep medication=2]	29.16	-3242.12	3300.44
[sleep medication=3]	0a		
[marital status =single]	10.78	-6365.13	6546.11
[marital status =divorced]	46.67	-1313.11	1334.69
[marital status =married]	0a		
[education=high]	10.78	-1782.53	1760.96
[education=low]	13.77	-1694.56	1667.00
[education=normal]	0a		
[drug=cannabis]	46.43	-4802.86	4709.98
[drug=alcohol]	54.98	-6972.26	6862.30
[drug=multiple drug use]	0a		
Adjusted for age, substance of use, marital status and education			
Link function: Logit. OR= odds ratio			
a. This parameter is set to zero because it is redundant.			

Supplementary table 3.

CATPCA - Principal Components Analysis for Categorical Data .

**Total Variance Explained (%) by each component**					
**1**	**2**	**3**	**4**	**5**	**6**
19.50	11.62	10.98	12.85	11.25	12.97

Component Loadings.

	**Dimensions**					
	**1**	**2**	**3**	**4**	**5**	**6**
Sleep medication	**0.893**					
Sleep disturbances	**0.743**					
Initiation insomnia	**0.609**					**0.480**
Sleep quality	**0.538**	**0.688**				
Cannabis use		**0.900**		**0.510**		
Alcohol use				**0.558**		**0.497**
Sleep efficiency			**0.845**			
Multiple substances use			**-0.798**			
Superficial sleep				**0.823**		
Sleep duration				**0.617**	**0.464**	
Daytime dysfunction					**0.855**	
Anxiety					**0.643**	
Substance use onset					**0.512**	
Maintaining insomnia						**0.898**

	Dimensions					
	**1**	**2**	**3**	**4**	**5**	**6**
Insomnia symptoms						**0.649**
Sleep latency		**0.486**		**0.439**		

Variable Principal Normalization. Dimensions and theirs loading factors. Only values higher then 0.4 were considered significant and presented.

CATPCA - Principal Components Analysis for Categorical Data.

	Dimension	
	1	2
Gender	-0.494	0.662
Main psychiatric diagnosis		0.848
Insomnia symptoms	0.718	
Anxiety severity	0.796	
Sleep Duration	0.832	
Marital status (a)		
Education(a)		

Variable Principal Normalization.

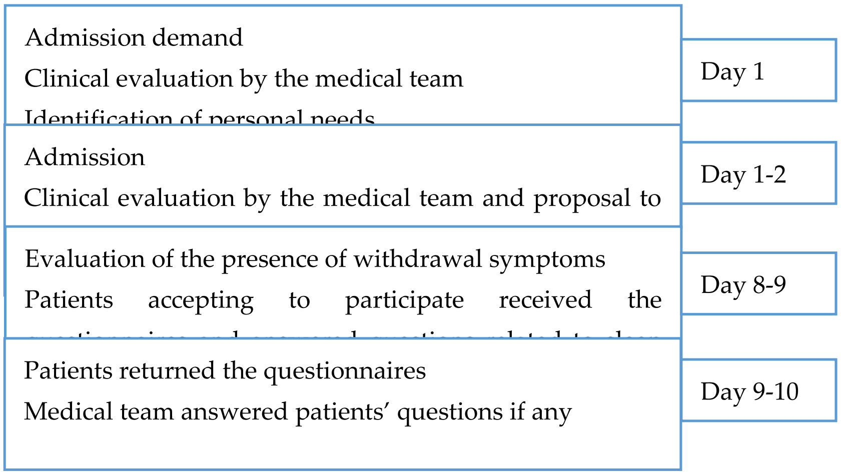

Supplementary figure 1. Time line of data collection
